# Context effects in language comprehension: The role of emotional state and attention on semantic and syntactic processing

**DOI:** 10.3389/fnhum.2022.1014547

**Published:** 2022-11-25

**Authors:** Dorothee J. Chwilla

**Affiliations:** Donders Centre for Cognition, Donders Institute for Brain, Cognition and Behaviour, Radboud University Nijmegen, Nijmegen, Netherlands

**Keywords:** emotion, attention, language comprehension, heuristic processing, N400 cloze effect, P600 effect, embodied theories, abstract symbol theories

## Abstract

Semantics and syntax are core components of language. The prevailing view was that processing of word meaning and syntactic processing happens in isolation from other systems. In light of proofed interactions between language and other systems, especially with perception, action and emotion, this view became untenable. This article reviews Event-related potential studies conducted at the Donders Centre for Cognition exploring the interplay between language comprehension and a person’s emotional state. The research program was aimed at an investigation of the online effects of emotional state on semantic processing and syntactic processing. To this aim we manipulated mood via film fragments (happy vs. sad) before participants read neutral sentences while their EEG was recorded. In Part 1, it is shown that mood impacts online semantic processing (as indicated by N400) and the processing of syntactic violations (as indicated by P600). Part 2 was directed at a further determination of the mechanisms underlying these interactions. The role of heuristics was examined by investigating the effects of mood on the P600 to semantic reversals. The results revealed that mood affects heuristic processing. The next step consisted of an assessment of the role of attention, in the mood-by-semantics and mood-by-syntax interaction. This was accomplished by recording EEG while manipulating attention via task next to emotional state. Participants performed a semantic or syntactic judgment task vs. a letter-size judgment task. The main ERP results were as follows: (i) attention interacts with the mood effect on semantic processing and syntactic processing, respectively, (ii) the effects of mood on semantic processing and syntactic processing are reliable, and (iii) the mood effects on semantic processing are not fixed but context-dependent. In Part 3 the effects of mood on the processing of script knowledge and general world knowledge are presented. Part 4 closes with a discussion of the mechanisms involved in the mood-by-language interactions and recommendations for future research. Regarding the underlying mechanism we propose that heuristics based on semantic expectancies or syntactic expectancies play a key role in the mood-by-language interactions. The results support the view that language takes place in continuous interaction with other (non-language) systems.

## Introduction

One of the most fascinating aspects of language is the speed at which readers or listeners are capable of understanding language. How can language users within a fraction of a second select the right words from a data base consisting of about 50,000 words stored in their memory, access their meanings and fit them into context? The efficiency and apparent ease with which language users pull off this incredible achievement led to the widespread notion that language happens automatically (see for a discussion: Hartsuiker and Moors, 2017). This holds par excellence for processing of word meaning and syntax. Moreover, the classic view is that language comprehension takes place in isolation from other systems like perception, motor action and emotion (Fodor, 1983). This modular view has been challenged by interactions of language with perception (e.g., Stanfield and Zwaan, 2001; Pecher et al., 2003; Amsel et al., 2014) language with action (e.g., Glenberg and Kaschak, 2002; Pulvermüller, 2005), and language with emotion (e.g., Havas et al., 2007; Niedenthal, 2007).^1^

With the advent of modern brain imaging techniques, in particular, functional magnetic resonance imaging (fMRI), it became possible to identify the neural circuits involved in cognition (memory, attention, and language) and emotion in the human brain. FMRI studies have shown that the prefrontal cortex plays a crucial role in cognition (see for a review on working memory: D’Esposito et al., 2000) and the amygdala plays an essential role in emotion perception and production (Davidson and Irwin, 1999). There is mounting evidence from studies investigating the interaction of cognition and emotion that these two systems are not categorically different but deeply interwoven in the brain (Damasio, 2005; Pessoa, 2008; Linquist and Barrett, 2012; Shackman et al., 2015). As proposed by Pessoa (2008, 2013) brain regions like the prefrontal cortex are not cognitive and regions like the amygdala are not emotional (see also Fuster, 2001; Shackman et al., 2009; Birn et al., 2014). Instead, these two brain regions are of central importance for the regulation of adaptive behavior and perform a variety of functions depending on the context and task environment. Based on these brain imaging results in this article it is assumed that the brain areas involved in cognition and emotion are strongly interconnected in the brain.

While fMRI is ideally suited to unravel the neural networks involved in the cognition-emotion interface its temporal resolution is limited. To acquire a fine-grained picture about the time course of cognition-emotion interactions, a technique with a high temporal resolution is needed. Event-related potentials (ERPs) have an exquisite temporal resolution at the level of milliseconds. For this reason, the ERP method has become a standard tool over the last 30 years to track the time course of cognitive processes in real time (Coles and Rugg, 1995). It has been especially crucial for understanding a dynamic process like language in which on average 3–5 words per second are processed for meaning and integrated into context (Kutas and Van Petten, 1988).

In this review, the main interest concerns the interplay between language and emotion. I report ERP studies that were aimed at investigating the time-course of interactions between language processing and emotion in real time. Specifically, I focus on the ERP work carried out in my group at the Donders Centre for Cognition, where we looked at possible interactions between processing of word meaning and emotion, and between syntactic processing and emotion.

In contrast to ERP studies in which the impact of emotional language (emotion words, content or prosody) on comprehension has been examined (e.g., Herbert et al., 2008; Martín-Loeches et al., 2012), we investigated the effects of a person’s emotional state on neutral language. A description of the effect of emotional language on language comprehension falls outside of the scope of this article (see for a review on this topic Hinojosa et al., 2019). Emotional states, in contrast to emotions, which are typically evoked by objects or specific events in the environment and are typically short-lived, often occur without awareness of an eliciting event and are less intense (e.g., Frijda, 1986). Of interest, here, emotional states—like being in a happy or sad mood—have been demonstrated to affect behavior, color perception, impact decision making and the scope of our attention (for a review see Clore and Huntsinger, 2007). As sketched below studying the effects of emotional state on language is of theoretical importance: it allows one to test classical modular theories against interactive theories of language. To separate these theories effectively, the use of neutral language is required. Because if emotion words are clustered in semantic memory^2^ then effects of mood can be accounted for not only by interactive theories, but also by classic (abstract-symbol) theories of language comprehension.

## Structure of this review

The central question of this research program was whether a person’s emotional state^3^ influences online sentence processing. The structure of this review is as follows: In Part 1, the effects of emotional state on the processing of word meaning (Study 1) and the processing of syntactic anomalies (Study 2) are presented. The main findings from these studies were that mood modulates the online processing of word meaning (as indicated by N400) and the processing of syntactic violations (as indicated by P600).

After having demonstrated interactions between mood and these key components of language, Part 2 is directed at a further determination of the mechanisms behind these mood-by-language interactions. The background is formed by three scenario’s which could have caused the mood-by-language interactions. Study 3 examined the role of heuristics in bringing about the mood-by-language interactions. The take-home message from this study was that mood modulates heuristic processes in language online. The next step was to elucidate the role of more general factors in the mood-by-language interactions. Specifically, the goal was to separate the effects of mood from those of attention on the processing of syntactic anomalies (Study 4) and the processing of word meaning (Study 5), respectively. These studies revealed that attention plays a role in the mood-by-language interactions reported in Studies 1 and 2 and hence that its effects have to be controlled for in future studies. In addition, these studies replicated the effect of mood on syntactic processing (Study 4) and semantic processing (Study 5).

In Part 3 work is presented in which the relation between mood and the processing of different kinds of world knowledge was examined. In Study 6 the effects of mood on the processing of more abstract script knowledge was investigated in a priming study. In Study 7 the effect of mood on the processing of more general world knowledge in sentence context was explored. The main outcome of these studies was that the processing of script knowledge was modulated by mood whereas the processing of more general world knowledge in sentence context was not. Part 4 summarizes the main results of this research program followed by a general discussion about the underlying mechanisms and recommendations for future work.

## Part 1

### Emotional state and semantic processing

The studies in this part will be presented in more detail because the results form the cornerstone of this review.^4^ The first study (Chwilla et al., 2011)—on the emotion-by-language interface—targeted on semantics. Meaning is a fundamental aspect of language. The sentence “He killed her” means something radically different from the sentence “He kissed her.” The difference lies in the meanings of the individual action verbs “kill” vs. “kiss.” The verb kill captures an act of violence leading to irreversible death while the verb “kiss” captures an action of tenderness reflecting affection. The goal of Study 1 was to investigate whether, and if so how, emotional state affects online processing of word meaning in reading. Before spelling out the experimental approach used to tackle this question the relevance of this endeavor should be explained.

Two theoretical perspectives regarding the representation of word meaning have been presented. According to abstract symbol theories, the brain is conceived of as an organ for building internal representations of the external world. A main assumption of these theories is that knowledge resides in a semantic memory system separate from the brain’s modal systems for perception (audition and vision), action (movement and proprioception), and emotion (Fodor, 1983). According to these theories, meaning arises from the syntactic combination of abstract, amodal (non-perceptual) symbols that are arbitrarily related to entities in the real world (e.g., Collins and Loftus, 1975; Masson, 1995; Anderson et al., 1997; Landauer and Dumais, 1997). For example, according to one of the most frequently cited theory of Collins and Loftus (1975), meaning arises from the pattern of relations among nodes in a network. On this view language understanding boils down to manipulating abstract symbols.

A different theoretical perspective about the representation of word meanings are embodied approaches to cognition (e.g., Lakoff, 1987; Glenberg, 1997; Barsalou, 1999; MacWhinney, 1999; Zwaan and Taylor, 2006). Here, the brain’s primary purpose is not to represent the external world but to regulate behavior. A key assumption of this approach is that mental processes such as thinking or language understanding are based on the physical or imagined interactions (Stanfield and Zwaan, 2001; Barsalou, 2008) that people have with their environment. The starting point of the *embodied* approach is that the structure of the body is very important in that it determines the range of effective actions. According to embodied views, meaning is based on our current interactions or previous experiences of interactions with objects in different kinds of environments. Current or past Body X Environment interactions guide us in how to think about, that is, simulate the perceptual and action details required by a situation. On this view comprehending sentences basically consists of simulating actions.

The two classes of theories differ in their predictions regarding the effects of emotional state on processing of word meaning. According to abstract symbol theories, processing of word meaning is performed by a central cognitive module—that is, separable from the systems for perception, motor action, and emotion. Therefore, activation of word meaning should be resistant to fluctuations in emotional context. In contrast, according to embodied theories, words are meaningful because they are grounded in perception, action, and emotion; this is where they derive their meaning from. Investigating the effects of emotional state on semantic processing, therefore, is of importance because the presence of language-by-emotion interactions would challenge abstract symbol theories and support embodied theories of meaning (Damasio, 2005; Glenberg et al., 2005; Havas et al., 2007; Niedenthal, 2007).

Following this logic, the aim of Study 1 was to test abstract symbol vs. embodied theories of meaning against each other by examining the effects of mood on online semantic processing. Because the focus was on semantics we zoomed in on N400, a temporal landmark of meaning processing (Kutas and Hillyard, 1980; for reviews see Kutas and Federmeier, 2000, 2011). N400 amplitude systematically varies with the degree of expectedness of a word in a given context; the higher the expectancy, the smaller the N400. A word’s predictability in context is typically assessed with the cloze task (Taylor, 1953). In this task a sentence fragment is presented and participants have to guess what the next word will be. The probability that a word is produced as a sentence continuation is named its cloze probability. N400 amplitude has been shown to be inversely correlated with cloze probability (Kutas and Hillyard, 1984) and modulations in N400 amplitude as a function of cloze value are referred to as the N400 cloze effect.

We tested abstract vs. embodied theories against each other by exploring whether the standard N400 cloze effect is modulated by mood. The experimental approach consisted of inducing different emotional states (a happy mood vs. a sad mood) while the EEG was recorded and participants were asked to read sentences part of which contained highly expected events (high-cloze sentences e.g., “In that library the pupils borrow *books*…”) and unexpected events (low-cloze sentences e.g., “The pillows are stuffed with *books*…”). To rule out task-related activity not part of normal language processing (Brown et al., 2000) participants were asked to read for comprehension. No additional task was given.

#### Mood induction

**FIGURE 1:**
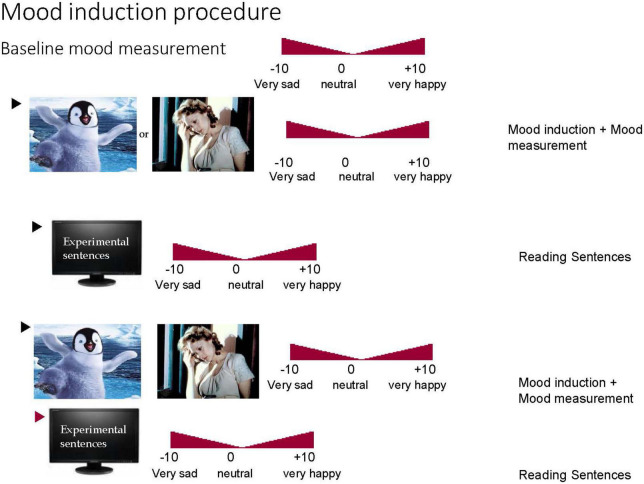
Visual sketch of the mood induction procedure.

In order to assess the effects of emotional state on language understanding, a necessary condition is that the intended mood states are successfully induced. In the studies reported here fragments from films were used for mood induction. It has been shown that films are a highly effective means to induce both a positive and negative mood (Westermann et al., 1996). Therefore, we manipulated a participant’s mood by presenting film fragments that either displayed fragments from a happy movie (fragments from Warner Brother’s movie “Happy Feet”) or from a sad movie (fragments from the movie “Sophie’s Choice” a second world war drama). The effectiveness of the mood induction in the present studies was assessed by collecting mood ratings before mood induction (baseline) and before and after each film clip (which were presented preceding the critical language materials) (see Figure 1). To prolong the induction of the intended mood several film clips were presented (for more information see Chwilla et al., 2011).

The predictions were as follows: Based on the ERP literature a standard N400 cloze effect was predicted. The crucial question was whether an interaction between emotional state and the N400 cloze effect would be observed. If so, a modulation of the N400 amplitude should occur in the standard N400 time window (within 300–500 ms following critical word onset).

The main results of Study 1 were: The mood induction was effective in inducing the intended emotional state. After watching happy film fragments, participants were happier than after watching sad film fragments. Similarly, after watching sad film fragments, participants were sadder than after watching happy film fragments. A significant difference in mood scores between the happy and the sad mood condition was present after each of the film clips. With these data it was possible to determine the effects of mood on online semantic processing.

**FIGURE 2:**
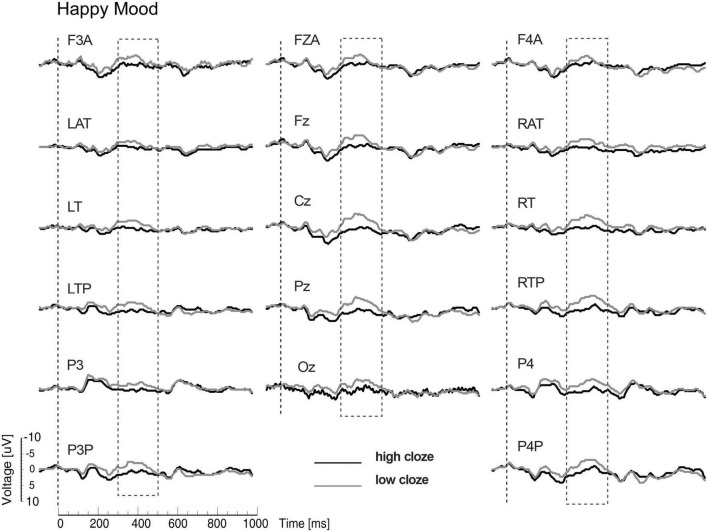
Modified Figure 2 from Chwilla et al. (2011). Study 1: Grand ERP waveforms for the happy mood condition, time-locked to the onset of the critical noun superimposed for the two levels of cloze probability (high, low) for all midline sites and a representative subset of lateral sites. N400 was measured in the time window from 300 to 500 ms. Negativity is plotted upwards.

**FIGURE 3:**
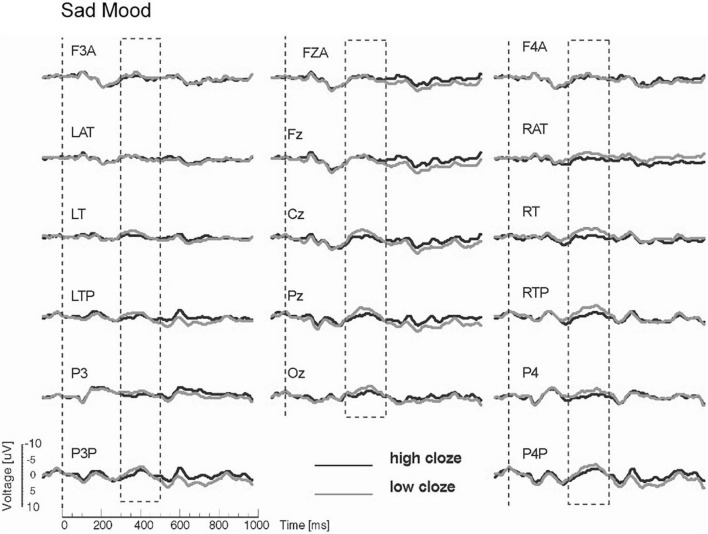
Modified Figure 3 from Chwilla et al. (2011). Study 1: Grand ERP waveforms for the sad mood condition, time-locked to the onset of the critical noun superimposed for the two levels of cloze probability (high, low) for all midline sites and a representative subset of lateral sites. N400 was measured in the time window from 300 to 500 ms. Negativity is plotted upwards.

The N400 results showed interactions between mood and cloze probability. Therefore, the ERPs are presented separately for the two mood conditions. The interactions reflected a strong reduction of the N400 cloze effect for the sad compared to the happy mood condition (see Figures 2, 3). In particular, the analyses for the midline sites revealed the presence of a clear N400 effect for the happy mood condition, but absence of an N400 effect for the sad mood condition. The absence of an N400 effect across the midline for the sad mood condition is remarkable, given that the centroparietal midline sites (Cz and Pz) typically show the largest N400 cloze effects. The interaction between mood and cloze probability for the lateral sites indicated that the N400 cloze effect for the happy mood condition showed a widespread bilateral distribution, including anterior, central, temporal, posterior and occipital sites. In contrast, for the sad mood condition, an N400 cloze effect was mainly limited to the right hemisphere.

Correlation analyses with the size of the N400 effect and mean mood rating as factors revealed strong correlations between the size of the N400 effect and the mood ratings for all central and posterior electrodes. These analyses indicated an increase in N400 cloze effect with increasing happiness for the happy mood condition. With correlations ranging from −0.65 to −0.79, between 42 and 62% of the variation in the size of the N400 effect, was accompanied by variations in mood. The results of the analyses supported the assumption that emotional state -for an important part -contributed to the effect.

The conclusion from Study 1 was that mood modulates online processing of word meaning. Importantly, the mood-by-cloze interactions for N400, support embodied theories of meaning and challenge abstract symbol theories of meaning according to which computation of word meaning is a *modular*---*context independent- process*: The demonstration of an emotion-context effect cannot be accounted for by abstract symbol theories.^5^

Which cognitive mechanism could underlie this effect? A potential answer comes from the emotion literature. Here, it is generally agreed upon that differences in mood lead to qualitatively different strategies and that mood-dependent processing styles exist (e.g., Schwarz, 2002). A positive mood leads to greater cognitive flexibility and a broader focus, relying less on the details of a situation and more on top-down schematic processing (Fredrickson, 1998). It validates accessible cognitions and leads to a more global, category level of processing of information to what is already known on the basis of our world knowledge including schemas and stereotypes (e.g., Kimchi and Palmer, 1982; Gasper and Clore, 2002). This processing strategy fits well with the finding of strong N400 context effects to high-cloze sentences representing highly familiar scenarios based on world knowledge (e.g., “picking flowers in a meadow”). Negative mood, in contrast, seems to focus our attention more narrowly on specific details of a situation: It invalidates accessible cognitions and fosters local, item-specific processing (e.g., Schwarz, 2002). In a review on the effects of emotion on cognition, Clore and Huntsinger (2007) propose that many famous phenomena in cognitive science such as semantic priming, global superiority effect, and false memories occur when people are in a positive mood but do not arise or occur in a reduced form, when people are in a negative mood. The strong reduction of an N400 cloze effect observed for negative mood was consistent with the view that people in a sad mood are less open to accessible cognitions of what happens in the world around them.

### Emotional state and syntactic processing

Next to semantics, syntax is a main component of language. More than this, syntax has a special status. This is indicated by the general belief that syntactic analysis has priority over semantic analysis as reflected by syntax-first models (Frazier, 1987). In line with this many language scientists assume that in the absence of syntactic uncertainty, semantic processing is always fundamentally dependent on the output of the syntactic processing system (see for a discussion: Kolk et al., 2003; Osterhout et al., 2008).

Regarding sentence processing syntax is crucial for thematic role assignment. Let us take a simple sentence “Bob kicked Colin.” Here syntactic information is required to figure out who does what to whom -that is, who does the kicking. Regarding the generality of mood effects on comprehension, it was important to investigate whether emotional state also impacts syntactic processes. The focus in Study 2 was on the P600: a centroparietally distributed slow positive shift peaking around 600 ms following critical word onset (see for a review; Osterhout et al., 2008). An increase in P600 amplitude has been reported to different kinds of syntactic violations amongst which subject-verb agreement violations, verb-inflection violations and phrase-structure violations. For Study 2, subject–verb agreement errors were chosen, because they have been shown to reliably elicit a P600 effect (see Vos et al., 2001).

In Study 2 (Vissers et al., 2010) we compared the ERP signatures to subject-verb agreement errors to their syntactically correct counterparts under different mood conditions (happy vs. sad mood). Sentences with or without subject–verb agreement, such as (2) were presented to participants in a happy mood and participants in a sad mood, (2) The parents who about their daughter talked [plural] (correct) (word-by-word translation); The daughter who about her parents talked [plural] (incorrect) (word-by-word translation). Note that the scenarios described in these sentences are equally plausible (parents talking about their daughter vs. daughter talking about her parents). A standard P600 effect was predicted for the syntactically anomalous sentences in response to the critical verb compared to the syntactically correct verb (e.g., Friederici et al., 1993). The question was whether the P600 effect to syntactic anomalies is modulated by mood. A happy vs. sad mood was induced in the same way as in Study 1. If mood alters processing of syntactic violations this should be indicated by a mood-by-syntactic correctness interaction for the P600 (measured in the 600 to 800 ms time window following critical verb onset).

**FIGURE 4:**
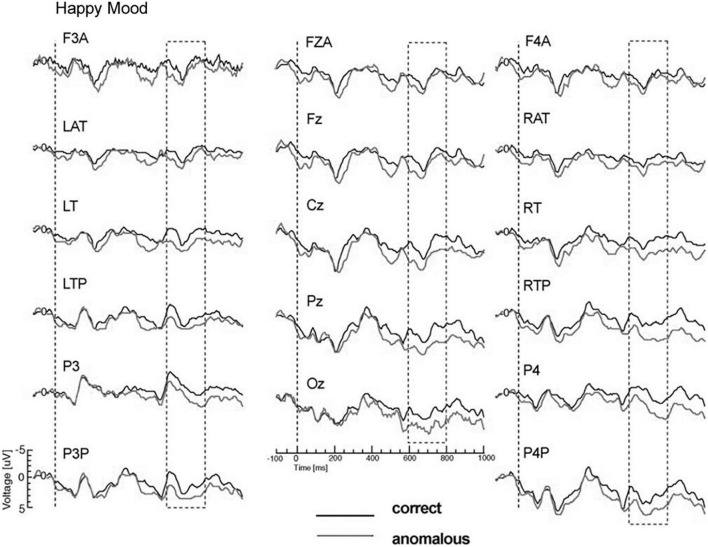
Modified Figure 2 from Vissers et al. (2010). Study 2: Grand ERP waveforms for the happy mood condition, time-locked to the onset of the critical verb superimposed for the two levels of syntactic correctness for all midline sites and a representative subset of lateral sites. The dashed rectangles indicate the time window (600–800 ms) in which P600 amplitude was measured. Negativity is plotted upwards.

**FIGURE 5:**
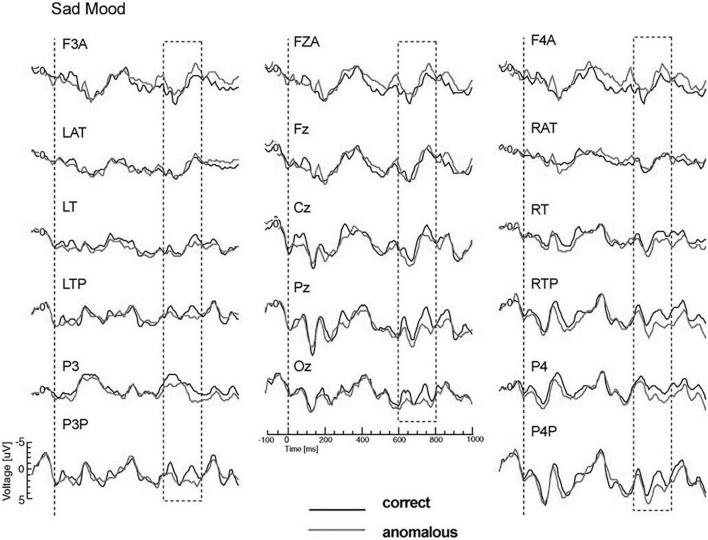
Modified Figure 3 from Vissers et al. (2010). Study 2: Grand ERP waveforms for the sad mood condition, time-locked to the onset of the critical verb superimposed for the two levels of syntactic correctness for all midline sites and a representative subset of lateral sites. The dashed rectangles indicate the time window (600–800 ms) in which P600 amplitude was measured. Negativity is plotted upwards.

The main findings were as follows: participants were happier after watching happy film fragments and sadder after watching sad film fragments. This means the mood induction was effective. For P600 a mood-by-syntactic correctness interaction was obtained. The interaction reflected a broadly bilaterally distributed P600 effect for the happy mood condition (see Figure 4) and a strong reduction in P600 effect for the sad mood condition (see Figure 5). For sad mood a P600 effect only occurred at two lateral posterior sites (P3p and P4p).

Correlation analyses (with the size of the P600 effect and mean mood rating as factors) confirmed that the observed changes in P600 effect were accompanied by changes in emotional state. For the happy mood condition these analyses (for sites with a significant P600 effect) indicated the happier the mood, the larger the P600 effect. For the sad mood condition these analyses disclosed the sadder the mood, the smaller the P600 effect.

This was the first report of a modulation of the standard P600 effect to syntactic violations by mood. After having demonstrated that the processing of subject-verb agreement errors is influenced by mood let’s turn to possible explanations of this finding.

#### Possible explanations for the mood-by-syntactic processing interaction

##### Scenario 1

Based on the sensitivity of the P600 to syntactic well-formedness, one possible explanation relates to syntactic factors: mood could selectively affect syntactic processing. As stated above, manipulations of syntactic structure are well-known to elicit a P600; the P600 effect is proposed to reflect syntactic processing as such (Hagoort et al., 1993), syntactic reanalysis (Osterhout et al., 1994; Friederici, 1995), or syntactic integration difficulty (Kaan et al., 2000). On this account, the smaller P600 effect for the sad as compared to the happy mood condition could reflect reduced syntactic processing in the sad condition or increased syntactic processing in the happy condition.

##### Scenario 2

This scenario comes forth from the fact that the P600 is sensitive to heuristics: it is reliably evoked by semantic reversals (Kolk et al., 2003; Vissers et al., 2007). In semantic reversals subject and object of semantically correct sentences are switched rendering semantically odd sentences. For example, “The mice that fled from the cat” (paraphrase of Dutch sentence) is changed into “The cat that fled from the mice” (paraphrase of Dutch sentence). While the first version of the sentence is semantically plausible as it is highly expected based on world knowledge (heuristic interpretation), the newly created version leads to the semantically highly unlikely event that “a cat is fleeing from mice.” Based on the fact that the semantic reversals are not syntactically ambiguous (both noun phrases could serve as the agent and the patient of the action expressed by the verb ending the relative clause) these P600 effects cannot be explained by syntactic factors (note this finding triggered the development of the monitoring hypothesis of P600 presented in Box 1). The modulation in P600 amplitude, therefore, has to reflect heuristic factors. Further empirical evidence for the sensitivity of the P600 to heuristic factors will be presented below. Hence, the second scenario is that mood selectively affects the use of heuristics. According to this scenario, the reduction of the P600 effect in the sad mood condition relative to the happy mood condition could reflect a reduced use of heuristics, whereas the increase in P600 effect for the happy mood could be due to an increased use of heuristics.

**BOX 1 A brief sketch of the Monitoring Theory of Language Comprehension of Kolk et al. (2003).**

The monitoring theory of language comprehension of Kolk and colleagues forms the theoretical background on which the present studies on syntactic processing build forth. To further the understanding of these underpinnings this theory is presented in a nutshell. The observation of a P600 instead of an N400 to—syntactically unambiguous—semantic reversal anomalies triggered the formulation of the monitoring hypothesis (see Kolk et al., 2003; Van Herten et al., 2005). In line with the view that, in addition to syntactic parsing, people also process heuristically (e.g., Ferreira et al., 2002) this unexpected finding was explained by assuming that for semantic reversals there is a strong bias toward the semantically plausible interpretation.

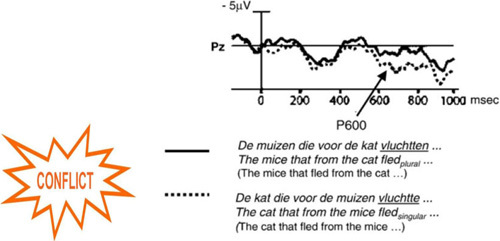

According to the monitoring hypothesis, when a strong expectation conflicts with what is actually observed, reanalysis is triggered to check the input for processing errors which is reflected by the P600. It is like asking yourself: Did I read that correctly? For the example depicted here, in semantic reversals, like ‘The cat that fled from the mice’ heuristics and syntactic algorithms produce different thematic interpretations. Whereas the heuristic based on world knowledge leads to the interpretation that the mice fled from the cat, the parsing routine leads to the interpretation that the cat fled from the mice. This conflict between the semantically plausible, expected thematic interpretation and the implausible thematic interpretation makes it necessary for the brain to re-attend the unexpected linguistic unit to verify its veridicality. On this view the function of the reanalysis reflected by P600 is not of a syntactic nature but of a more general nature.

Follow up research demonstrated that a conflict between an expected representation based on heuristic processing and an unexpected representation triggers a P600 effect at the sentence level, the word level and even at the conceptual level (Vissers et al., 2008). What is the locus of the conflict? Is the conflict resolved within or outside of the language system? In an fMRI study the same Left Inferior Frontal Gyrus activity observed in the classical stroop interference task was also found for various linguistic conflicts (Van de Meerendonk et al., 2011). This supports the claim that the locus of the conflict is outside of the language system. Monitoring is a process of executive control to guarantee quality of behavior and we have shown that language comprehension is not a purely automatic process, but in need of executive control.

Relevant for this review, these EEG results support a heuristic account of the P600 (see for a review, Van de Meerendonk et al., 2009 and Kolk and Chwilla, 2007). Note that a presentation of the different functional views on P600 falls outside of the scope of this review.

##### Scenario 3

Another explanation relates to the fact that P600 is also influenced by more general factors. In particular attentional factors, often varied by task demands, have been shown to affect P600 (e.g., Coulson et al., 1998; Kuperberg, 2007): an increase in attention coincides with an increase in P600 amplitude (effect). In other words, mood could modulate language comprehension by increasing or decreasing more general processes, like attention. More specifically, participants in a happy mood could pay more attention to the linguistic input, while people in a sad mood could pay less attention to the linguistic input.

The main conclusion from Study 2 was that the online processing of syntactic anomalies was affected by mood. If one assumes that P600 is an index of syntactic processing, this finding speaks against the modular nature of syntactic processes. However, as we argued, the mood effect on P600 could also be accounted for by heuristic factors or by more general factors. Important for the present deliberation, the results reveal that the effects of mood are not limited to semantic processing but generalize to the processing of syntactic violations.

## Part 2: Mechanism(s) behind the language-by-emotional state interactions

### The role of heuristics

In Study 3 (Vissers et al., 2013) we assessed the role of heuristics in mediating the effects of mood on language, testing scenario 2. Heuristic processing in language implies that language users do not always take into account all relevant information, in particular, both syntactic and semantic information. In line with this, Ferreira (2003) proposed that language processing is often based on a shallow representation of the input yielding a merely “good enough” rather than a detailed linguistic representation of an utterance’s meaning. Therefore, Ferreira and colleagues (e.g., Ferreira et al., 2002; Ferreira and Patson, 2007) have claimed that current models of language are missing an architectural component that can explain cases in which people exploit strategies or engage in heuristic processing of sentences that may then give rise to an inaccurate interpretation. Several lines of evidence support the existence of heuristic processing in language. So, Barton and Sanford (1993) have shown that most participants do not notice an anomaly in a sentence like “Bury the survivor.”

The aim of Study 3 was to further ascertain the locus of the effects of emotional state on sentence comprehension by investigating the effects of mood on the processing of semantic reversals, in which heuristics play a key role. This was accomplished by comparing the effects of mood on the P600 effect to semantic reversals (e.g., “The fox that at the poachers hunted [singular] stalked through the woods” (literal Dutch translation; paraphrase: “The fox that hunted [singular] the poachers.”) with the P600 to their plausible counterparts (e.g., “The poachers that hunted the *fox*…”). In the reversal sentence ‘the fox that hunted the poachers’ there is only one option: fox is the Agent, and poachers is the Theme’ rendering this sentence *syntactically unambiguous*. The materials and method for mood induction (happy vs. sad mood) were the same as those in Studies 1 and 2. The main question was whether the P600 to syntactically unambiguous semantic reversals would be modulated by emotional state. If so this would support the view that heuristic factors contribute to the mood effects on language comprehension. The underlying logic is, that given the fact that the semantic reversals are syntactically unambiguous, P600 effects to these anomalies cannot be explained in terms of syntactic processing and, therefore, have to reflect heuristic factors.

**FIGURE 6:**
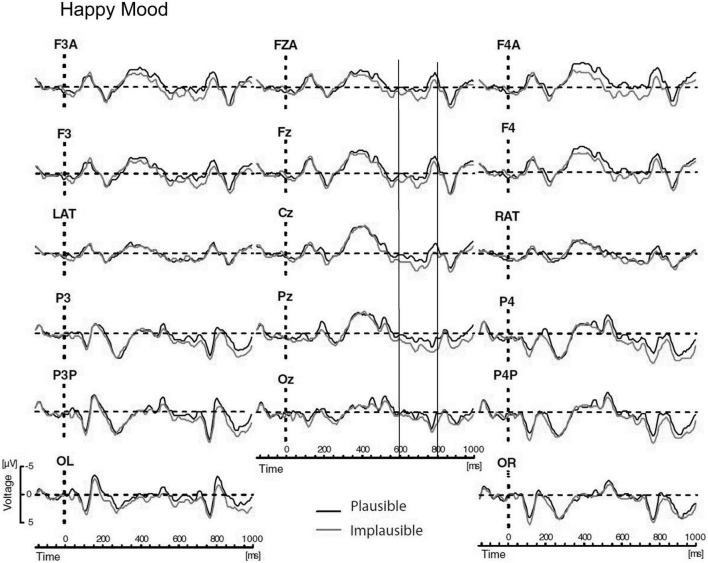
Modified Figure 2 from Vissers et al. (2013). Study 3: Grand ERP waveforms for the happy mood condition, time-locked to the onset of the critical verb superimposed for the two levels of condition for all midline sites and a representative subset of lateral sites. P600 was measured in the time window from 600 to 800 ms. Negativity is plotted upwards.

**FIGURE 7:**
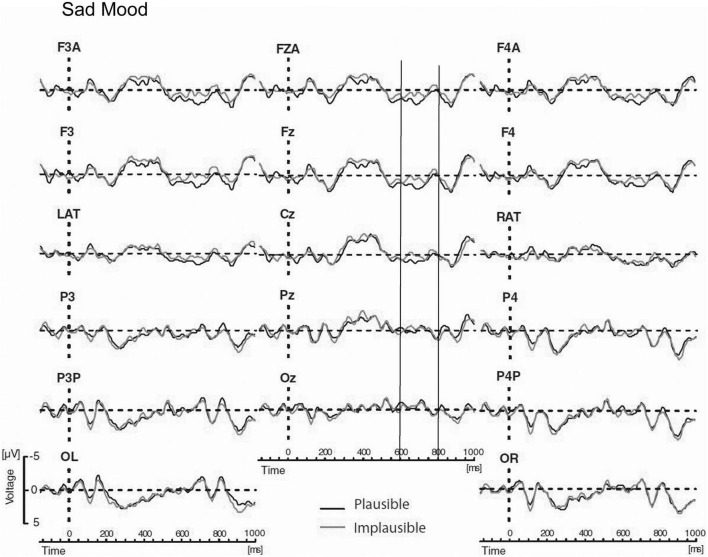
Modified Figure 3 from Vissers et al. (2013). Study 3: Grand ERP waveforms for the happy mood condition, time-locked to the onset of the critical verb superimposed for the two levels of condition for all midline sites and a representative subset of lateral sites. P600 was measured in the time window from 600 to 800 ms. Negativity is plotted upwards.

The main results of Study 3 were as follows: Again, the mood manipulation was effective. For P600 a mood-by-semantic plausibility interaction was obtained. The interaction indicated a broadly bilaterally distributed P600 effect for the happy mood condition vs. absence of a P600 for the sad mood condition (see Figures 6, 7). Correlation analyses confirmed that changes in P600 in happy mood were accompanied by changes in emotional state: the happier the mood the larger the P600 effect.

Regarding the locus of the effect, it is of relevance to determine similarities and differences of the mood effect on the processing of syntactic violations vs. semantic reversals. In Study 2, the correctness-by-mood interaction for syntactic anomalies indicated a large P600 effect for happy mood and a strong reduction in P600 effect for sad mood. In Study 3, the plausibility-by-mood interaction for the semantic reversals reflected the presence of a P600 effect for the happy mood vs. absence of a P600 for the sad mood. Thus, a similar but non-identical pattern of P600 effects as a function of mood was found for syntactic anomalies vs. semantic reversals. To directly compare the effect of mood on the two types of anomalies, global between-experiment-analyses for the P600, with mood and anomaly type (syntactic anomaly vs. semantic reversal) as between-participant factors and condition (correct vs. incorrect in Study 2 and plausible vs. implausible in Study 3) as within-participant factor were carried out. If there are reliable differences in the effect of mood on these two kinds of anomalies then a mood x anomaly type x condition interaction should be obtained. The global analyses yielded strong effects of condition and condition by mood interactions, confirming different P600 patterns for happy mood vs. sad mood across studies. More importantly there was no hint for a three-way mood x anomaly type × condition interaction. The results of the global analyses, therefore, support the notion that mood affected the processing of the syntactic P600 effects and the semantic P600 effects in a similar way. From this we concluded, that heuristics could have mediated the mood effect on processing of syntactic anomalies reported in Study 2.

But how can the use of heuristics impact the processing of syntactic anomalies? Note that syntactic errors in written language are very rare.^6^ In line with this, the assumption has been made that language users have a high expectancy to read syntactically correct sentences (Coulson et al., 1998; Vissers et al., 2010). On this account, the reduction of the P600 effect to syntactic anomalies in the sad mood condition could reflect a reduced use of heuristics. While the increase in P600 effect for the happy mood could reflect an increased reliance on shallow processing—that is, on a good enough representation of the linguistic input. The predicted P600 pattern is in line with a study from Vissers et al. (2007) that investigated the sensitivity of the P600 to heuristic processing using semantic reversals. According to the monitoring theory of language comprehension the P600 to semantic reversals reflects a conflict between the outcome of a plausibility heuristic with that of a parsing routine (see Box 1). In this study the use of heuristics was manipulated by instruction. Participants were told that semantic anomalies were created on purpose and that they should not be misled by these anomalies but instead focus on syntax or sentence structure. The main result from this study was that the focus-on-syntax instruction resulted in a strong decrease in P600 effect. This finding shows that expectancies play a major part in the generation of the P600. Regarding the direction of the effect, this study reveals that the use of heuristics typical for a positive mood enlarges the P600 effect. This scenario goes along well with the widely accepted notion of mood-dependent processing styles in the emotion literature.

### More general factors: The role of attention

In all studies reported so far a decrease in ERP effect for the negative mood compared to the positive mood was observed (N400 effect in Study 1 and P600 effect in Studies 2 and 3). This prompted the question whether more general factors like attention or motivation did contribute to the mood-by-language interactions observed to semantically unexpected items (Study 1 and 3) and syntactic anomalies (Study 2). Of special interest here is attention, because this factor has been shown to influence both N400 and P600. Regarding the N400, it has been established that this brain potential is sensitive to attentional manipulations, especially, task demands with larger N400 amplitudes or larger N400 effects with an increase in attention (e.g., Bentin et al., 1993; Chwilla et al., 1995). As already pointed out, attentional factors also affect the P600 (e.g., Gunter and Friederici, 1999). Therefore, the next step was to scrutinize the role of attention in the language-by-mood interactions, testing scenario 3.

#### Emotional state, attention, and syntactic processing

In Study 4 (Verhees et al., 2015) we explored the relation between attention, mood and the processing of syntactic anomalies. The *attention hypothesis* entails that participants in a happy mood could pay more attention to the linguistic input—here to syntactic violations—while people in a sad mood could pay less attention to the linguistic input. The proposal that happy mood would lead to greater attention aligns with claims in the emotion literature that positive emotions lead to attentional broadening (e.g., Fredrickson, 2001). This hypothesis was tested by studying the combined effects of mood and attention on online syntactic processing as tapped by the P600. On the assumption that attention and mood do affect the P600, the question was whether the effects of emotional state and attention on the P600 are additive and independent or whether they interact. In other words, if mood modulates the P600, is this modulation then a true effect of emotion or is it influenced by more general factors like attention? Mood was manipulated in the same way as in Studies 1 to 3. As in Study 2, sentences with subject-verb agreement errors and correct sentences were presented. Attention was manipulated by varying task demands, in particular by drawing attention either to syntactic features or to purely physical features of the words comprising the sentences. In the syntactic task, participants had to judge the syntactic correctness of the sentence. In the physical task, they had to indicate whether the sentence contained a word that differed in letter size. Note that the response had to be given to the sentence final word and *not* to the critical word. The probability of encountering a syntactic error and a syntactically correct sentence was 50%. In the syntactic judgment task, all words were presented in uppercase letters. In the letter-size judgment task, all words in the experimental sentences were presented in uppercase letters, whereas all filler sentences contained a word in lowercase letters. The physical deviation was only positioned in the filler sentences to avoid a confound by comparing a single violation in the syntactic judgment task with a double violation in the letter-size judgment task (i.e., an incorrect verb that was in a deviant letter size).

To sum up, in Study 4 the factors emotional state (happy vs. sad) and task (syntactic vs. letter-size judgment) were crossed. This design allowed a determination of the relative role of the factors mood and attention in the processing of syntactic anomalies. The predictions were as follows: Based on the literature we predicted a P600 effect to syntactic anomalies as well as a task effect with larger P600 effects for the syntactic judgment task than for the letter-size judgment task (Gunter and Friederici, 1999). Second, if the mood effect on P600 reported in Study 2 is robust then it should be replicated (with a reduction in P600 effect for the sad mood compared to the happy mood). If so, the question can be addressed whether attention contributes to the effect of emotional state. If attention contributes to the mood effect on P600, this should be reflected in an interaction between emotional state, task and the P600 effect of syntactic correctness. On the other hand, if attention does not contribute to the mood effect on P600, no such three-way interaction should be obtained. In the latter case an effect of emotional state and/or an effect of attention should be found on P600, in the absence of an interaction.

The main results were as follows: Again the mood induction was effective in eliciting a happy vs. sad mood allowing us to study the interplay between mood and attention when processing syntactic anomalies. For P600 an interaction between emotional state, task and syntactic correctness was found. The interaction indicated that the standard P600 effect to syntactic anomalies was modulated by attention in addition to emotional state.

**FIGURE 8:**
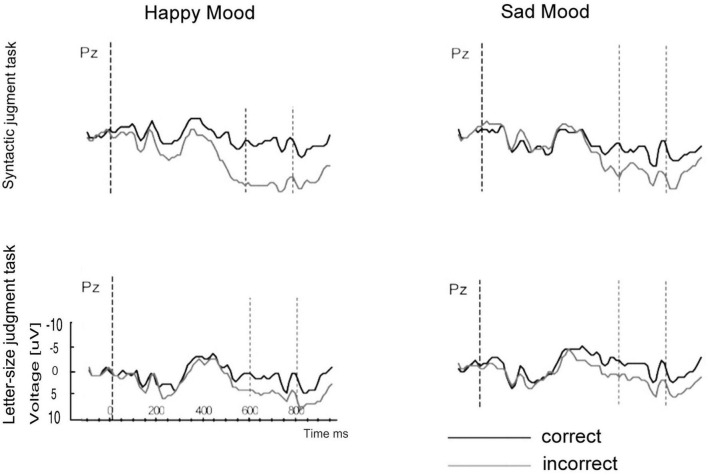
Modified Figure 8 from Verhees et al. (2015). Study 4: Grand ERP averages at Pz for both mood and task conditions.

**FIGURE 9:**
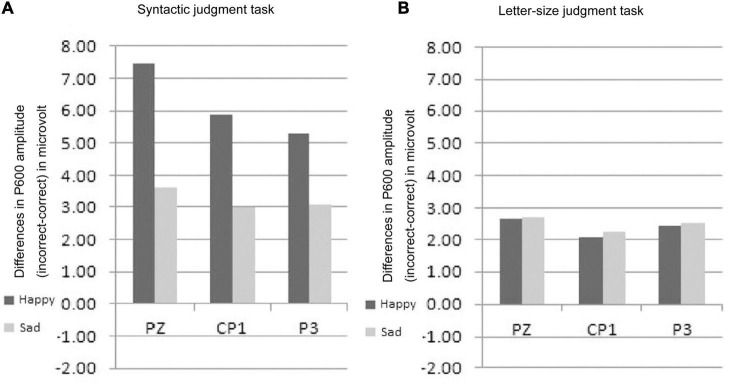
**(A,B)** Modified Figure 6 from Verhees et al. (2015). Comparison of the mean P600 amplitudes (incorrect-correct) for the happy and sad mood condition **(A)** for the syntactic judgment task and **(B)** for the letter-size judgment task.

In particular, the three-way interaction reflected that emotional state only affected P600 in the syntactic task and not in the letter-size judgment task (see Figure 8). When attention was directed at syntactic features, the mood manipulation led to a reduction in the P600 for the sad mood as compared to the happy mood. In contrast, when attention was focused on purely physical features, no difference between mood conditions was observed. What was the influence of attention on the P600 effect to syntactic violations? The focus of attention only had an impact on the P600 in the happy mood and not in the sad mood. Specifically, the attention manipulation led to a reduction in P600 when attention was directed at physical features (letter-size judgment task, see Figure 9B) compared to syntactic features (syntactic task) for the happy mood (see Figure 9A).

From Study 4 we drew two conclusions: First emotional state does affect syntactic processing. Again, a reduction in P600 effect occurred for the sad mood compared to the happy mood, this time in a syntactic judgment task. This means that the mood effect on the P600 to subject-verb agreement errors observed for reading in Study 2 is a reliable finding. In line with the results of Study 3, we propose differences in the reliance on heuristics, in this case syntactic expectancies, as the mechanism underlying these mood-related changes. A second and novel finding is that attention interacts with the online effect of mood on syntactic processing. Therefore, future studies researching the mood-by-language interface have to control for effects of attention.

#### Emotional state, attention, and semantic processing

The next step was to investigate whether more general factors like attention also play a role in the mood-related modulation of semantic processing as reflected by the N400. Here the *attention hypothesis* entails that participants in a happy mood could pay more attention to word meaning than people in a sad mood. Specifically, the reduction in N400 for the participants in the sad mood (who watched the sad film fragments) could reflect that they are preoccupied with processing of the drama and, hence, pay less attention to word meanings than participants who watched the happy film fragments. To test this hypothesis, Study 5 was conducted to separate possible effects of attention from those of emotional state on the N400 cloze effect (Chwilla and Tromp, 2013). The experimental approach was very similar to that of Study 4. Attention was manipulated via task (semantic vs. letter-size judgment task), and, in addition to, emotional state (happy vs. sad mood). In the semantic task, participants were asked to judge whether the sentences were semantically plausible. In the letter-size judgment task, they were asked whether the sentence contained a word that differed in letter size. As in Study 4 the response had to be given to the sentence final word and not to the critical word. The experimental sentences consisted of 50% high-cloze sentences (e.g., “In that library the pupils borrow books…”) and 50% low-cloze sentences (e.g., “The pillows are stuffed with books…”). To avoid a double anomaly in the letter-size judgment task (i.e., a semantically unexpected word in a deviant letter size [lowercase]) the physical deviation was always positioned in the filler sentences. Here the effects of emotional state and task were crossed to allow an assessment of the relative contribution of the factors mood and attention on the online processing of word meaning.

The predictions were as follows: First, in line with the literature we predicted an N400 cloze effect as well as a task effect, with strongly reduced N400 effects for the letter-size judgment task compared to the semantic task (Chwilla et al., 1995). Second, if the mood-related N400 modulation reported in Study 1 is reliable then the mood effect should be replicated (with a reduction in N400 effect for sad mood relative to happy mood). An investigation of the reliability of the in Study 1 observed N400 effect was also important because in one other study (Federmeier et al., 2001) a different effect of mood was reported—that is, a decrease in N400 for a (mildly) positive compared to a neutral mood. The key question was whether the effects of mood and attention are additive or interactive. If attention influences the mood effect on N400 an interaction between emotional state, task and the N400 cloze effect should be found. On the other hand, if the effects of mood and attention on N400 are independent then no such interaction should be obtained. If so, a main effect of the factor emotional state and/or attention should be found for the N400, without an interaction.

**FIGURE 10:**
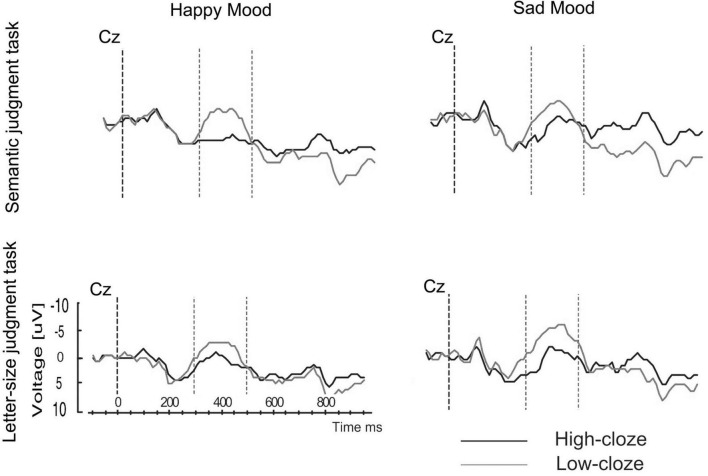
Study 5: Grand ERP waveforms at Cz for both mood and task conditions (Chwilla and Tromp, 2013).

The major findings from Study 5 were as follows: The mood induction was effective in eliciting a happy and a sad mood. Importantly, for N400 a three-way interaction between emotional state, task and cloze probability was present. The interaction indicated that the N400 cloze effect was modulated both by mood and attention. In particular, the interaction reflected that for happy mood an N400 effect occurred in the semantic task but was (statistically) absent in the letter-size judgment task. This means that people in a positive mood displayed the typical task effect reported in the literature. The pattern, however, differed for the negative mood. People in a sad mood showed an N400 effect both in the semantic task and in the letter-size judgment task. Moreover, as Figure 10 shows for the sad mood there was no sign of a reduction of the N400 effect in the letter-size judgment task relative to the semantic task.

**FIGURE 11:**
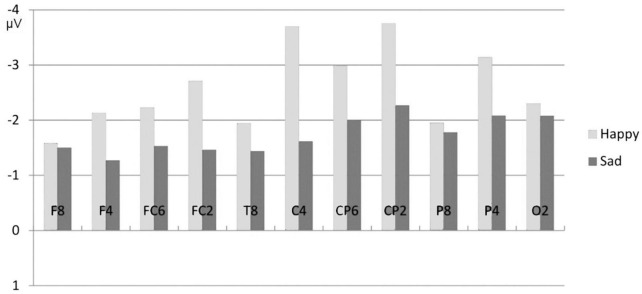
Study 5: Comparison of the mean N400 amplitudes (implausible-plausible) for the happy and sad mood condition, for the semantic task for the electrodes of the right hemisphere (Chwilla and Tromp, 2013).

How did attention affect the modulation of the N400 effect by mood? Focusing attention on the semantic level reduced the influence of mood on the N400 cloze effect compared to Study 1 (in that the number of electrodes showing N400 effects for the sad mood in Study 5 was increased). However, a mood effect again was found as indicated by the fact that the N400 effect was significantly smaller for the sad mood than for the happy mood (see Figure 11). In other words, the N400 mood-by-semantics interaction—reported in Study 1—was replicated.

This was the first study investigating the joint effects of mood and attention on the standard N400 cloze effect. The observed three-way interaction reveals that the N400 cloze effect was influenced both by emotional state and by attention (varied via task demands). The task-related N400 modulation observed for the happy mood—a large N400 effect for the semantic task vs. absence of an N400 effect for the letter-size judgment task—converges with previous N400 results comparing a deep with a shallow processing task (e.g., Chwilla et al., 1995). Opposite to this, for the sad mood condition there was no reduction in N400 cloze effect for the letter-size judgment task compared to the semantic task. In other words, while for the happy mood the occurrence of an N400 cloze effect was limited to the semantic task, for the sad mood a clear N400 effect occurred regardless of the task. Thus, opposite to the results of Studies 1–4, *no decrease* in ERP response (N400 and P600, respectively) occurred for the sad mood relative to the happy mood. Here, a reduction and even absence of an N400 effect for the letter-size judgment task was obtained for the happy mood and not for the sad mood. The importance of this finding of a reversed pattern for the sad relative to the happy mood (in the letter-size judgment task a clear N400 effect in the sad mood but not in the happy mood) is that it demonstrates that sad mood does not lead to a general attenuation of semantic processing.

The ERP results from Study 5 warrant two major conclusions: First, sad mood does not yield an overall reduction in processing word meanings in sentences. Conversely, consistent with the emotion literature a happy mood vs. sad mood leads to qualitative differences in language comprehension (Schwarz, 2002; Vissers et al., 2013). A second chief conclusion bolstered by the present findings is that the effects of mood are not fixed but depend on the context (see also Huntsinger et al., 2014). I will come back to this in the discussion.

## Part 3. Emotional state and the processing of world knowledge

The ERP studies reported so far show that processing of word meaning at the sentence level is influenced by mood. We have taken these results to accord well with the emotion literature in which it is generally agreed upon that there are mood-dependent processing styles (Kimchi and Palmer, 1982; Gasper and Clore, 2002). People in a positive mood are biased toward exploiting world knowledge and process information at a more global relational level. In contrast, people in a sad mood process information more analytically, pay attention to details, have a more narrow focus and, hence, are less inclined to exploit their world knowledge. On the assumption that specific cognitive processing styles are characteristic for people in a happy mood vs. sad mood, one would predict differences in their sensitivity to exploit diverse kinds of world knowledge.

In Study 6 (Menn and Chwilla, 2017) we examined how one specific kind of world knowledge, namely script knowledge, is processed by people in a happy mood vs. sad mood. As described in Chwilla and Kolk (2005), “Scripts, also referred to as schemata refer to knowledge structures or sets of expectations build on past experience that have been conceived as the building blocks of cognition (e.g., Barlett, 1932; Rumelhart, 1980). Schemata are mental representations of stereotypical situations. A famous example is the restaurant script of Schank and Abelson (1977). A script for a restaurant involves the actors, props, entry and exit conditions, and action sequences like sitting at a table, ordering food from a menu, and drinking wine.” Here we tested for differences in exploiting script knowledge as a function of mood using a variant of the semantic priming paradigm. Similar to the decrease in reaction time reported when a word is preceded by a related word compared to an unrelated word (e.g., when the word “DOCTOR” is preceded by the word “NURSE” vs. “PILOT”), a decrease in N400 amplitude is found when a word is preceded by an associatively and/or semantically related word compared to an unrelated word. Analog to the reaction time priming effect this is referred to as the N400 priming effect. It has been shown that an N400 priming effect also occurs to words that are related in a more abstract way, as is the case for scripts (Chwilla and Kolk, 2005).

The goal of Study 6 was to investigate whether the N400 priming effect observed for script knowledge is modulated by mood. This was investigated by presenting word triplets that formed a conceptual script based on world knowledge (e.g., DIRECTOR—BRIBE—DISMISSAL or NAKED—SHY—TOWEL; but were not associatively and/or semantically related. To rule out associative relations between the 3 words free-association norms were collected for prime 1, prime 2 and the target. A triplet was rejected if the first, second, or third word was produced as an associate. The second criterion was that there was no semantic category relation between any of the three words. In addition, only word triplets were selected for which there was no (obvious) overlap in perceptual and/or functional features (for further details see: Chwilla and Kolk, 2005). The two prime words (here “DIRECTOR” and “BRIBE”) were presented simultaneously at central vision for 400 ms to the left and right of a fixation cross. The target word (here “DISMISSAL) immediately followed Primes 1 and 2 (Inter-stimulus interval was 0 ms). The participants were asked to judge the plausibility of the scenario depicted in the word triplets based on their knowledge of the world. EEG from 36 female participants was recorded and a happy vs. sad mood was induced using our standard induction procedure. Based on the previous study we predicted an N400 priming effect to script-related triplets compared to unrelated triplets. The main question was whether emotional state modulates the processing of script knowledge. If so, an interaction between mood and the N400 script priming effect should be found.

**FIGURE 12:**
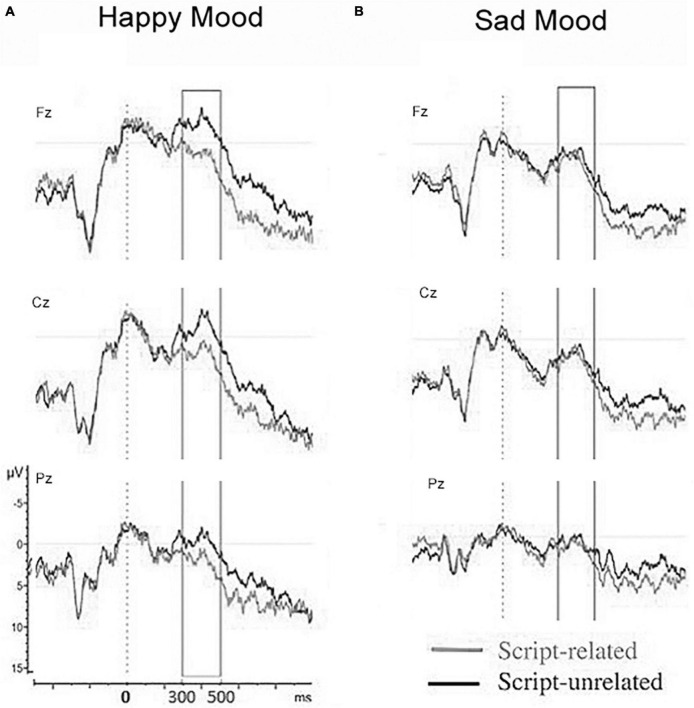
Grand ERP waveforms for the midline sites to the critical word of the script-related vs. unrelated triplets separately for the happy mood condition **(A)** and the sad mood condition **(B)** (Menn and Chwilla, 2017).

The main results of Study 6 were as follows: The mood induction was effective in inducing a happy and sad mood. As predicted for N400 a script priming effect was present in the standard time window (300–500 ms after target onset). Importantly, for N400 a mood-by-plausibility interaction was obtained. The interaction indicated the presence of an N400 script priming effect for the happy mood, but absence of an N400 effect for the sad mood (see Figures 12A,B), respectively. In addition, correlations between emotional state and N400 were found: The more positive the mood the larger the N400 script priming effect.

The novel finding from Study 6 was that processing of more abstract script knowledge is modulated by emotional state: An N400 effect to conceptual scripts occurred in a happy mood but not in a sad mood. The results of the correlation analyses further bolstered the assumption that mood influences the processing of script knowledge. One might ask on which grounds we base the claim that these word triplets tap script knowledge? For this we used a co-occurrence measure, namely Latent Semantic Analysis (LSA) of Landauer and Dumais (1997). LSA has been shown to be a sensitive technique for detecting even subtle differences in relatedness between words when traditional methods like free association or semantic relatedness judgments suggest that the items are unrelated (Chwilla and Kolk, 2002). Pairwise comparisons between prime 1 and prime 2, prime 1 and the target, and prime 2 and the target were carried out for the related items and the unrelated items. The LSA analyses yielded significantly higher semantic similarity values for the script-related triplets than for the unrelated triplets. The results confirmed that the scripts present familiar, and in that sense old well-known contexts, and therefore are readily available based on our notions of what kinds of objects and events make up the world.

What did we learn from Study 6? Consistent with the emotion literature, this ERP study provides evidence for differences in processing style as a function of emotional state. Happy mood biases toward top-down processing and facilitates exploiting script knowledge. In contrast, a sad mood biases toward bottom-up processing and discourages exploiting script knowledge. In addition, this study revealed that mood effects are not limited to the sentence level—but for more abstract script knowledge—can be observed at the word level.

The next step was to investigate whether mood affects the processing of more general world knowledge at the sentence level. Our brains are big warehouses in which huge amounts of familiar knowledge are stored (e.g., Kutas and Federmeier, 2000). This knowledge encompasses a multitude of things amongst which facts like, for instance, the capital of countries and more general knowledge about objects and events that occur in the world around us. An example of the latter would be, that you give a rose to a lover and not a cactus. Hagoort et al. (2004) were the first to show—using N400—that world knowledge is immediately integrated into context—that is, within the same time frame, as the integration of word meaning. A good example of their materials is “Dutch trains are *yellow* and very crowded” vs. “Dutch trains are *white* and very crowded.” Trains can have either color but it’s the knowledge about trains in the Netherlands that trains are usually yellow.

In Study 7 (Chwilla, 2018) we used the sentence materials from the Hagoort et al. study^7^ to address the question whether emotional state impacts processing of general world knowledge. This was accomplished by inducing a happy vs. sad mood and studying the effects on the processing of words that were either consistent with people’s general world knowledge (WKC) or inconsistent with world knowledge (WKI). Another example of the materials: WKC: “The writer Shakespeare wrote many **sonnets** and plays.” WKI: “The writer Shakespeare wrote many **melodies** and plays.” As in the original study we added selection restriction violations as a semantic control condition. Based on the Hagoort et al. study, we predicted—next to a standard N400 effect to semantic violations—an N400 effect to WKI critical words compared to WKC ones. The results from Study 7 were as follows: The mood induction was effective in inducing the intended moods. For N400 a graded pattern was observed across moods: N400 amplitude was largest to semantic violations, significantly reduced for world knowledge violations and significantly smaller for words fitting general world knowledge. In brief we replicated the results from Hagoort et al. (2004). Important for the present purposes, for N400, no mood-by-condition interaction was found. These results suggest that the processing of general world knowledge violations is not influenced by emotional state. In other words, the mood effect reported for semantic anomalies in Studies 1 and 5, and for script knowledge in Study 6, does not seem to extend to the general world knowledge violations at the sentence level. It should be pointed out that the absence of a mood effect on the processing of world knowledge violations could also be due to a power problem. Further work is needed to check the reliability of this null effect. Another possible explanation for this unexpected result in terms of differences in cloze probabilities between studies is provided below.

## Part 4: General discussion

### Overview of the main findings

What did the present research program reveal about the relationship between processes of language comprehension and a person’s emotional state?

In Study 1 we tested abstract symbol theories against embodied theories of language comprehension against each other by determining the effects of mood on the processing of word meaning. The mood-by-cloze interaction for the N400 calls into question that processing of word meaning is performed by a central cognitive module. The results of Study 1, therefore, support embodied theories of language comprehension and pose a challenge for abstract symbol theories of word meaning.

In the field of psychology recently more attention has been paid to the replicability of experimental findings. With this in mind it is not trivial to point out that the replication of the mood effect on the N400 cloze effect in Study 5 demonstrates the robustness of the mood-by-semantic processing interaction. The contribution of attention to the N400 cloze by mood interaction further calls into question that activation of word meaning reflects a modular process. It appears that processing word meanings when reading neutral sentences involves a continuous interaction of the language system with other non-language systems, here with the systems of emotion and attention.

Study 2 indicates that the effects of mood are not limited to semantics but generalize to the processing of syntactic violations. What are the theoretical consequences of the P600 mood-by-syntactic correctness interaction? If one adheres to the view that the P600 indexes syntactic processes then the interaction could be taken as evidence against modular views of syntactic processing. In fact, the absence of a mood-by-syntactic processing interaction for P600 to subject-verb agreement errors has been presented as support for a more modular nature of syntactic processing (see Van Berkum et al., 2013). However, due to the fact that P600 is also sensitive to other factors than syntax alone, we argued that there are at least two alternative explanations for this interaction: one in terms of heuristic processing and one in terms of more general factors, like attention.

In Study 3 the role of heuristics was explored by investigating the effects of mood on semantic reversals. The mood-by-semantic plausibility interaction reported for the P600 supports the view that heuristics play a major role in the emotion-by-language interactions. The similarity of the mood-related P600 pattern for syntactic violations and semantic reversals was pointed out. This finding is in line with our claim that heuristics gave rise to the mood-by-syntax interaction reported in Study 2.

In Study 4 the effects of attention^8^ —manipulated via task—in addition to the effects of mood on the processing of syntactic violations were investigated. The mood-by-task-by-syntactic correctness interaction revealed that attention contributes to the mood effect on the P600. For people in a happy mood the typical task effect was found -that is, a P600 effect occurred when the attention was directed at syntactic features but not when attention was directed at physical features. While the P600 was overall reduced for the sad mood condition, for sad mood, no difference in P600 effect between tasks was found. Regarding the effect of mood on the P600: The P600 effect to syntactic violations for the syntactic task was significantly larger for the happy mood than for the sad mood. In other words, the mood-by-syntactic correctness interaction for P600 reported in Study 2 was replicated showing the robustness of this experimental finding. This is an important result because some researchers proposed that the P600 is mainly insensitive to fluctuations in emotional state (Jimenez-Ortega et al., 2012; Van Berkum et al., 2013).^9^ The fact that attention interacts with mood when processing syntactic violations underscores the necessity to give up the notion that language occurs in isolation and instead view language as an interactive phenomenon.

In Study 5 the joint effects of attention and mood on the N400 cloze effect were examined. For N400, a mood-by-task-by-cloze interaction was obtained. The mood effect on N400 was replicated for the semantic task: as in Study 1, the N400 amplitude was significantly reduced for the sad mood compared to the happy mood. The attention manipulation had a different effect on the two mood conditions: people in a happy mood showed the typical task effect -that is, a reduction of the N400 effect in the letter-size judgment task compared to the semantic task. In contrast, people in a sad mood showed an N400 effect *regardless* of the task. The importance of this finding lies in the fact that this was the first study showing a reduction—and even absence of an N400—for the happy mood and *not* for the sad mood. This result clearly contradicts the claim that the reduction in N400 effect for the sad mood as compared to the happy mood, reported in the present studies reflects a general decrease in language processing. This proposal accords well with the vast amount of evidence in the emotion literature that differences in emotional state go together with *qualitatively* different processing styles. At the same time the observed N400 pattern in Study 5 reveals the effects of mood are not fixed but dependent on the context. This flexibility in cognitive processing style—here observed for positive mood as a function of task—has been discussed by Huntsinger et al. (2014). As they point out “despite decades of research demonstrating a dedicated link between positive and negative affect and specific cognitive processes […] the relation between affect and cognition is not fixed, but instead, is highly malleable.” p = 600. The fact that we found no task effect for the negative mood, could indicate that people in a sad mood compared to those in a happy mood are less flexible in allocating their attention to different aspects of the language input. Apparently sad people tend to stick to a local bottom-up strategy when processing word meanings.

### Heuristic account of the mood-by-language interactions

#### Semantic expectancies and syntactic expectancies

We have interpreted the mood effects on semantic processing and syntactic processing as reflecting the operation of heuristics. In Studies 1 and 3 the heuristics were based on semantic expectancies triggered by a mismatch with highly familiar scenarios based on world knowledge (e.g., “pillows stuffed with books” in Study 1 and “mice chasing cats” in Study 3). As pointed out heuristics can also be based on syntactic expectancies especially when encountering syntactic violations (as in Studies 2 and 4) which in written text are very rare. According to the P600 monitoring hypothesis of language perception first proposed by my colleague Herman Kolk (see Box 1), heuristic processing comes into play when encountering syntactic errors in the following way. When reading syntactically incorrect sentences based on heuristic processing the expected syntactically correct interpretation of these sentences is accessed. Based on the expectation for a syntactically correct sentence a conflict will be triggered when readers encounter an ungrammatical inflection. We propose that a conflict between a syntactically correct, expected representation and a syntactically incorrect, unexpected representation of the verb triggers reanalysis to filter out possible processing errors which is reflected by the P600.

That expectancies in reading *per se* have a powerful influence on P600 has been demonstrated by Coulson et al. (1998). They manipulated the probability of syntactic violations in visually presented sentences. When the probability of encountering a syntactic error was low (20%) a P600 effect occurred to syntactic violations, whereas when the probability of sentences containing a syntactic error and correct sentences was reversed (80% syntactic errors) a P600 effect occurred to syntactically correct sentences. Therefore, linking the mood-related changes in P600 amplitude to processes of expectancy is in line with probability effects on this brain wave.

The only result that seems inconsistent with the view that expectancies play a prominent role in mediating the mood effects on language comprehension comes from Study 7. If processes of expectancy are the driving force why then no interaction between mood and processing of general world knowledge for N400 was found? One factor that likely played a role is cloze probability. In Studies 1 and 5 which yielded N400 mood-by-language interactions, the cloze probability of the sentences was very high (>90%). Inspection of the sentences of Study 7 unveiled that the cloze probability of the critical items rendering the sentence consistent with general world knowledge varied (between 0 and 100%). For the Shakespeare example above, for instance, the cloze probability of “The writer Shakespeare wrote many *sonnets*” was 19% while the cloze probability for the train example was 44%.

Given that cloze probability is one of the main determinants of the N400 amplitude these differences in expectancies between studies most likely have affected the results. To engage processes of prediction overall a high(er) cloze probability of the sentences could be required. To clarify this issue future studies should systematically vary the cloze probability of the sentence materials and study its effect on the mood-by-language interaction.

In this article cloze probability has been used as a measure of semantic expectancy, more specifically of a word’s predictability in a given context. This is standard practice in the field of psycholinguistics. For the validation of the materials we used the original version of the cloze task (Taylor, 1953) in which a sentence fragment is presented in written form and participants are asked to guess what the next word will be. The probability that a word is produced as a sentence continuation is referred to as its cloze probability. In light of the fact that the importance of prediction in human information processing (Bar, 2007) and language processing has been recognized, more recently (Clark, 2013; Kuperberg and Jaeger, 2016; Verhagen et al., 2018; see for electrophysiological evidence Federmeier, 2007; Van Petten and Luka, 2012), the use of the cloze task has gained great momentum. Relevant for the present discussion, some researchers present a different view on the cognitive process underlying cloze probability. Staub et al. (2015) claim that “the process of producing a cloze response is best understood as an activation-based race process, what subjects are actually doing in the cloze task is reporting the first word that reaches a threshold level of activation.” On this account words with a high-cloze probability are easier to process because they are more strongly activated (e.g., DeLong et al., 2005). If cloze probability reflects the relative degree of lexical activation then what in the literature has been referred to as predictability effects could in fact reflect contextual activation effects. This raises the question whether the semantic expectancy effects reported here—and taken to reflect heuristic processing—could be accounted for by a “word’s relative level of pre-activation”?

From the ERP literature it is clear (see Kutas and Federmeier, 2011 for a review) that N400 is sensitive to both prelexical processes (spreading activation, expectancy) and postlexical processes (integrative mechanisms). Based on this the possibility that the semantic expectancy effects could reflect activation processes cannot be excluded.^10^ Note the results of Study 6 speak against the view that the mood effects are mediated by a word’s relative level of pre-activation. This is the case because as pointed out before the script-related triplets (e.g., DIRECTOR---BRIBE---DISMISSAL) were *not* associatively and/or semantically related (no category relation and/or overlap in perceptual or semantic features between the words) but exclusively conceptually related. In addition, the words did not elicit each other in free association, neither when presented alone, nor when the two primes were presented together. Consequently, the mood effect on the N400 script priming effect cannot be accounted for by differences in the pre-activation level of the words. This implies that these mood effects were caused by a postlexical (integrative) mechanism and not by a prelexical mechanism (spreading activation^11^ or expectancy processes). Hence, the mood effect on the N400 effect to script knowledge also cannot be accounted for by expectancy processes. Facilitation of exploiting more abstract world knowledge, dependent on the specific circumstances, can take place either via processes of forward prediction or via postlexical integration processes. Integrative mechanisms have been shown to operate very rapidly, most likely automatically (Chwilla et al., 2007) and to occur independently of the direction of the semantic relation which reveals that they are highly flexible (Chwilla et al., 1998). As Pickering and Gambi (2018) emphasize in their review on prediction in language comprehension, a clean demarcation of processes of prediction from those of integration often is very difficult. As they point out it is particularly hard to find unequivocal empirical support for prediction because the large majority of effects presented as evidence for prediction is also compatible with integration.^12^ For an experimental approach to address this issue the reader is referred to this review. Note that ERP research on the processing of novel meanings also forms an effective way to separate spreading activation and expectancy processes from integration mechanisms (see Chwilla et al., 2007; Chwilla, 2012).

There is a second dataset contradicting that a word’s pre-activation level plays a part in mood effects on language comprehension. In a priming study (Chwilla, 2019) we did not find evidence for a mood effect on the N400 priming effect to associatively and/or semantically related word-pairs. Even for strongly bidirectionally associatively related items which elicited each other in free association (e.g., ‘‘spider’’---‘‘web’’) ---and yielded large N400 effects---no mood effect was found. Furthermore, if a word’s pre-activation is critical in bringing about the mood effects then such an effect would be predicted for unidirectionally forward-related pairs (e.g., ‘‘stork’’---‘‘baby’’^13^) and not for unidirectionally backward-related word-pairs (e.g., “baby”—“stork”). This is the case because according to the most frequently cited spreading activation theory of Collins and Loftus (1975) activation spreads forward from the prime to the target and not in backward direction. There was no indication, however, for a mood effect on semantic priming, neither for strongly bidirectionally related word-pairs relative to unrelated pairs nor for unidirectionally forward related items compared to unidirectionally backward related items. Also based on the results of this study it is considered unlikely that word pre-activation is a relevant factor in the language-by-mood interactions. This leads to a recommendation for future research: In order to separate the contribution of prelexical mechanisms from postlexical mechanisms in mediating mood effects in language, a comparison of N400 effects for unidirectionally forward related materials with N400 effects for unidirectionally backward related materials, can be of great value (see McNamara, 2005).

A question that still needs to be addressed is how a heuristic account can explain the context-dependency of the N400 mood effect reported in Study 5. The traditional view is that expectancy processes reflect a more controlled process the operation of which is dependent on factors like the task (e.g., Brown et al., 2000), composition of the materials, and the time people have to generate predictions (e.g., Neely, 1977). Though the automatic vs. controlled nature of prediction processes is currently under debate (e.g., Kuperberg and Jaeger, 2016), the absence of an N400 effect for the letter-size judgment task observed for the happy mood could reflect that processing at the meaning level is required to engage prediction processes.

### Effects of mood at different levels of the language system?

In this review the effects of mood on different language aspects at the sentence level were exposed. It would be relevant to know whether mood impacts processes at other levels of the language system.

Does mood influence processing at the word level? The results for RT are mixed. Storbeck and Clore (2008) reported a mood effect on the RT semantic priming effect. In contrast with this, Maire et al. (2017) propose that mood does not affect processes of lexical access in a lexical task.

Aligning with this we did not find a mood effect on the RT priming effect or N400 priming effect even for strongly bidirectionally related word-pairs (Chwilla, 2019).

It appears that the impact of mood on language comprehension mainly occurs at higher levels of the language system. This assumption fits with a study from Havas et al. (2007) in which the effects of mood at the sentence and word level were compared. They induced different emotional states by manipulating facial expressions (“forcing” participants to smile vs. blocking their smile) and examined the effects on the comprehension of emotionally valenced sentences and emotion words in lexical decision. Facial expression influenced the processing speed of emotionally valenced sentences, but did *not* affect processing speed in lexical decision. The latter result even held for strong emotion words. Based on this Havas and colleagues, adheres of embodied theories, argued that “simulation using emotional systems is predominantly a sentence- or phrase-level phenomenon” p. 439. The fact that we observed a mood effect on the N400 effect to script knowledge may indicate that effects of emotional state only occur for more abstract types of information.

What about mood effects beyond the sentence level? A good example comes from Egidi and Nusbaum (2012) who studied the crossed effects of mood (happy, sad, and neutral) and the valence of sentence endings (positive and negative) on semantic processing during discourse comprehension. An increase in the N400 amplitude was found for a mismatch between the valence of the sentence and mood, relative to the match condition. This indicates that the mood effect for semantic processing generalizes to the discourse level. Van Berkum et al. (2013) investigated the effects of mood on the processing of verb-based expectancies. For example, *‘‘Peter annoyed Mary because *he*’’* vs. *‘‘Peter annoyed Mary because *she**.*’’* In the first sentence there is a high expectancy for the pronoun, which is not the case for the second sentence. They showed that mood impacts referential anticipation. This was indicated by a positivity---in the 400--600 ms epoch following the critical word---to bias-inconsistent pronouns compared to bias-consistent pronouns. This positivity was found for the happy mood but not for the sad mood. Van Berkum et al. interpret these results as reflecting that readers in a happy mood make predictions, in this case, about a specific person, while readers in a sad mood refrain from doing so.^14^ Therefore, it looks like expectancies at the discourse level also play a part in bringing about mood effects in language. Based on these findings I recommend to study mood-by-language interactions at the sentence level or beyond.

### Other outstanding questions

#### Investigation of sex differences in the effects of mood on language

The participants in this research program consisted of female students. The reason for this was that research has shown that sex influences the functioning of the human brain, including emotional memory (see Cahill, 2006, 2010). In line with this, results from Federmeier et al. (2001) suggested that female and male participants process meaning differently in a positive vs. neutral mood. Because the majority of psychology students at Radboud University consists of females we chose this population for the present research. It will be important to systematically assess similarities and differences between sexes in the effects of mood on language processing.

##### Mood induction: Externally vs. internally generated emotional states

In the studies presented mood was manipulated by showing film fragments displaying scenes from a happy movie or a sad movie. The choice of the mood induction procedure was based on a study from Westermann et al. (1996). These investigators compared 11 methods for mood induction and concluded that movies and stories are very effective in inducing positive and negative emotional states. So far, the film fragments worked well in inducing both a happy mood and a sad mood. However, one may wonder to what extent externally generated emotions are qualitatively similar/different from internally generated emotional states? This is an important question. Note that mood induction procedures are available which have been shown to reliably yield internally generated positive and negative emotional states. One example is the Autobiographical Emotional Memory task, in which participants are asked to write about remembered emotional memories associated with the corresponding emotions (Strack et al., 1985). Future studies could use this or similar techniques to address this issue.

A somewhat related query is to what extent the emotions people are experiencing in the lab are similar or different to those experienced in daily life? A challenge for future research is to move research from the lab to more realistic emotion settings. Some labs have started to use virtual reality devices in combination with the ERP method in an attempt to shed light on this issue (see e.g., Hofmann et al., 2021).

##### Lack of a neutral mood condition

In the studies reported here no neutral mood condition was included. The reason for this was that it was difficult to find materials suited for induction of a neutral mood condition. Likewise, the question of what constitutes a proper neutral baseline in psycholinguistic research is a matter of debate (Brown et al., 2000). Therefore, we have chosen to constrain our research to investigating the effects of a positive (happy) mood and a negative (sad) mood. A task for future researchers, therefore, consists of constructing a valid neutral baseline condition and compare the effects of a neutral mood with that of a positive mood and negative mood. This can help to infer whether positive mood strengthens the ERP components or the negative mood reduces them, or both.

#### Hemispheric specialization: A link to the brain

The visual half-field paradigm is a standard technique and effective tool for investigating hemispheric differences (e.g., Hunter and Brysbaert, 2008). A large body of research supports the assumption that the two cerebral hemispheres may be differentially involved in the regulation of affect (see for a review: Davidson, 1995). A study from Kutas and Federmeier (2000) nicely illustrates how the visual half-field paradigm can be applied in an ERP study to test for hemispheric differences in online semantic processing. Based on the N400 results of this study they claim that the two cerebral hemispheres play different parts in sentence comprehension. In particular, they argue that the left hemisphere is biased toward prediction processes while the right hemisphere is mainly involved in integration processes and refrains from making predictions. It is interesting to mention that in Study 1 differences in N400 scalp distribution were observed as a function of mood. While for the happy mood a broad bilateral distribution of the N400 effect was observed, the N400 effect for the sad mood was mainly restricted to the right hemisphere. This pattern of results was taken to suggest that people in a happy mood use both a predictive and an integration strategy while people in a sad mood only seem to exploit an integration strategy. Use of the visual half-field paradigm in future studies provides a unique opportunity to tease apart the (initial) contribution of the left cerebral hemisphere vs. the right cerebral hemisphere to the mood-by-language interactions.

#### How does mood affect language processing in the auditory domain?

Up to now, research on the effects of mood on language processing has been mainly conducted in the visual domain. Studies employing rapid serial visual presentation of stimuli (like we did) face the criticism that this presentation format is quite different from how people process language in ordinary life. While the N400 and P600 have been shown to reliably occur across modalities it will be vital to clarify whether the mood effects reported here for the visual domain will also generalize to the auditory domain.

#### Effects of mood on language production?

Whether mood also impacts different processes of language production has so far hardly been investigated. The first studies exploring this exciting topic have been taken to indicate that emotional state, in particular a negative mood, impacts language production processes, in particular, phonological encoding (Hinojosa et al., 2017). A second study indicated that inclusion of taboo words in a picture-word interference task, changes the effect of non-taboo emotion words during speech production (White and Abrams, 2021). Expanding the horizon to more natural dialogue, recent results suggest that mood can influence the alignment in referring expressions produced in an interactive context (Out et al., 2020). This new promising line of research is still in its infancy. Gaining insight into the effects of mood on language production will also help to build a bridge between the domains of language comprehension and production.

## Conclusion

The mood-by-language comprehension interactions reported in this review demonstrate that emotional state reliably modulates semantic processing and the processing of syntactic violations. These interactions are of theoretical relevance because they challenge classical modular theories and support interactive theories of language comprehension. In addition, this research has shown that attention contributes to the mood-by-language interactions observed for semantics and syntax. For processing of syntactic violations, again a decrease in P600 effect was found for the sad mood compared to the happy mood, this time in a syntactic judgment task. In contrast, for semantic processing, a reduction in N400 cloze effect as a function of task was found for the happy mood but not for the sad mood. For the sad mood a clear N400 cloze effect occurred regardless of task. This shows that the effects of mood are not fixed but context-dependent. Clearly more studies in which the effects of mood and attention are crossed are required to foster our understanding of the interplay between language, mood and attention for semantic and syntactic processing. These findings underscore the importance of incorporating contextual factors, in particular of mood and attention, into theoretical frameworks for affective neurolinguistics (see Kissler, 2019).

At a more general level, our results contribute to the, by now, large body of literature in language and other domains that call into question a clean demarcation between the language system and the brain’s modal systems for perception, action and emotion. This view fits well with fMRI studies that show that emotion and cognition (memory, attention, language) strongly interact in the brain. When studying language it is important to realize that language processes are not isolated processes, but are in continuous interaction with other (cognitive) processes. Regarding the effects of attention, it has been demonstrated that attention also plays a main role in language production (Roelofs and Ferreira, 2019). As sketched above, whether and if so how, mood impacts language production processes has to await future research.

## Author contributions

The author confirms being the sole contributor of this work and has approved it for publication.

## Footnotes

1 Note that attempts to replicate some of the classical findings in the embodied language literature, including some original evidence for the existence of mental simulation in language comprehension (Zwaan and Pecher, 2012) and the action-compatibility effect (Glenberg and Kaschak, 2002) have failed (Papesh, 2015; Morey et al., 2022).

2 Bower (1981) presented an associative network theory of emotion and memory. According to this theory emotion words are stored in an associated network with the mechanism of spreading of activation facilitating access to related nodes. To separate the contribution of the different mechanisms to the emotion-by-language interactions the use of neutral language materials, therefore, is important.

3 Here the term emotional state is used interchangeable with mood.

4 Note that excerpts from the original articles reviewed here are integrated in this review. It concerns the following articles: Vissers et al. (2010, 2013), Chwilla et al. (2011), and Verhees et al. (2015).

5 As pointed out by one of the reviewers, the fact that classic abstract symbol theories cannot account for the N400 mood effect on semantic processing in Study 1, does not necessarily imply that modality-specific representations—as proposed by embodied theories—are needed to explain the effect.

6 For instance, Garnham et al. (1981) found a total number of 191 slips of the tongue (including grammatical speech errors) in a text corpus of 200,000 words; this is no more than about one speech error per thousand words.

7 I am grateful to Lea Hald for providing us with the sentence materials and the cloze probabilities of these items.

8 Note that for Studies 2, 3, 4, and 5 additional analyses were also performed for early attentional components, in particular for the P1 and the N1 (125–175 ms and 175–225 ms time windows measured from critical word onset). The P1 and N1 have been shown to reflect perceptual and attentional processing, respectively (Mangun and Hillyard, 1991). The results of these additional analyses for the P1 and N1 did not affect the results of the later ERP effects (N400 or P600) with respect to the mood-by-semantics or mood-by-syntax interactions.

9 Note that Jimenez-Ortega et al. (2012) did observe a mood effect on another syntax-relevant ERP component, the Left Anterior Negativity (LAN). The LAN has been proposed to reflect syntactic processes (e.g., Hahne and Friederici, 1999) or processes of working memory (e.g., Coulson et al., 1998). In this study mood was manipulated by presenting positive, negative or neutral text paragraphs preceding a sentence that contained a syntactic anomaly. The main result was that the P600 was not affected by mood while the LAN was modulated by mood. Some methodological differences, between this study and Study 2 for example, the type of mood induction, the language materials and/or language studied hamper a direct comparison of the ERP results across studies. One aspect that complicates the interpretation of the ERP results of Jimenez-Ortega et al. is that the effectiveness of the mood manipulation has not been assessed. In line with this the authors suggest that the discrepancy in P600 effects between their study and the Vissers et al. (2010) study, could be due to the fact that the emotional paragraphs might not have been effective in inducing the intended mood. The reliability of the mood effect on the LAN, therefore, still needs to be demonstrated. Relevant for the present article we did not observe a LAN or a modulation of an early negativity by emotional state in the present studies.

10 For the pro’s and con’s of a lexical access account vs. semantic integration account on N400 see Kutas and Federmeier (2011).

11 We proposed that the mechanism of (automatic) spreading activation shares core characteristics with processes of lexical access while the mechanism of postlexical integration shares core characteristics with semantic matching (Chwilla et al., 1995; see also Lau et al., 2008). Insight into the contribution of these different mechanisms to the N400 effect, therefore, is helpful in linking the N400 to the processes of lexical access and integration.

12 For examples of N400 studies how to separate the processes of expectancy from those of integration in the semantic priming paradigm, by manipulating the proportion of related word pairs and/or time interval, the reader is referred to: Brown et al. (2000) and Lau et al. (2013).

13 A unidirectionally forward-related word-pair indicates that the word “stork” elicits the word “baby” in a free association task whereas the word “baby” does not elicit the word “stork” in free association.

14 Note that in the same study no effect of mood on the processing of subject-verb agreement errors was found.
